# Prevalence of Antidepressant Prescription in Adolescents Newly Diagnosed with Depression in Germany

**DOI:** 10.3390/children11101246

**Published:** 2024-10-16

**Authors:** Nimran Kaur, Corinna Doege, Karel Kostev

**Affiliations:** 1 Epidemiology, IQVIA, Bangalore 560 103, India; 2 Department of Pediatric Neurology, Center of Pediatrics and Adolescent Medicine, Central Hospital Bremen, 28205 Bremen, Germany; 3 Epidemiology, IQVIA, 60549 Frankfurt am Main, Germany; 4 University Clinic, Philipps-University, 35043 Marburg, Germany

**Keywords:** adolescents, antidepressants, depression, cohort study, Germany

## Abstract

Background: Depression is the most common mental illness in the world, found in nearly three in ten adolescents globally. This study aims to evaluate the prevalence of antidepressant prescriptions and the types of antidepressant therapy administered among adolescents diagnosed with depression in Germany. Methods: This retrospective cohort study, based on data provided by 30 child and adolescent psychiatrists, included adolescents aged 13–17 years with an initial diagnosis of depression between 2010 and 2022 (index date) documented in the IQVIA^TM^ Disease Analyzer database. Kaplan–Meier curves were used to investigate the one-year cumulative incidence of antidepressant prescriptions stratified by age, sex, and depression severity. Multivariable Cox regression analyses were used to assess the association between age, sex, depression severity, co-diagnoses, and antidepressant drug prescription. Results: A total of 6338 adolescents (mean age: 16 years, 67% female, 59% with moderate depression) were available. The cumulative incidence of antidepressant prescriptions was 61% and increased with age from 13 years old to 17 years old. Fluoxetine was the most prescribed drug, followed by Sertraline, Escitalopram, Serotonin and Norepinephrine reuptake inhibitors, herbal medications, and Mirtazapine. Obsessive–compulsive disorder and eating disorders were found to be significantly associated with antidepressant prescriptions within the spectrum of co-diagnosed conditions. Conclusions: Higher age, depression severity, and a co-diagnosis of an obsessive–compulsive disorder or eating disorder were significantly positively associated with antidepressant prescriptions in adolescents. Fluoxetine was the most frequently prescribed drug for depression.

## 1. Introduction

Depression is one of the most prevalent mental illnesses in the world [1]. A recent systematic review and meta-analysis concluded that nearly three in ten adolescents (10–19 years) globally suffer from depression. Consequently, the risk of developing clinical depression is greater among adolescents (13–18 years) than among young adults (>18–25 years) [2]. While an increase in depression was observed among 18–79-year-olds in Western countries, data from Germany indicated no change in its prevalence from 1997–1999 versus 2009–2012 in the same age group [3]. However, a study based on national outpatient claims data mentioned that the number of patients diagnosed with depression in Germany rose consistently from 2009 to 2017, and adolescents (15–19 years) presented with the greatest growth over this period.

A repeated cross-sectional study (2018–2019 and 2019–2020) from schools in 13 federal states of Germany observed higher levels of physical complaints specifically among 13–18-year-old girls in the post-pandemic wave [4]. In addition, a large survey showed that women were twice as likely as men to receive a confirmed diagnosis of depression between 2009 and 2017. A rise in the prevalence of depression, however, was more substantial in younger men (40%) than in women (20%), especially in adolescents aged 15–19 years (95%) as compared to older men aged 20–25 years (72%) [5]. Consequently, the Robert Koch Institute, Germany recommended conducting regular trend and cohort studies to assess the mental health status of young children during and beyond the COVID-19 pandemic [6]. Hence, research focusing on this particular age group with accurate diagnosis is required to enable researchers to understand the trends/presentation of depressive symptoms.

Plus, the diagnosis of specified depression symptoms has increased over time and is most pronounced for moderate depressive disorders, comprising one-third of all depression cases. Adolescents and young adults presented with the maximum relative increase in the proportion of patients suffering from moderate and severe depression when compared to other age groups [5]. Consequentially, major depressive disorders among children and adolescents can significantly impact their educational and/or vocational outcomes, interpersonal relationships, teenage parenthood, criminal convictions, disrupted physical and mental health, poor overall well-being, social isolation/withdrawal leading to suicidal ideation, suicide attempts, and eventually untimely death by suicide [7]. Though international studies have popularly researched the “epidemic” of mental disorders, the results have yielded conflicting evidence. As a result, studies are urgently needed to offer insight into the reasons behind these augmenting proportions in adolescents, backed up by legitimate and tangible evidence.

Currently, antidepressants are standard intervention strategies for numerous mental health conditions. Indeed, antidepressants for the treatment of child and adolescent psychiatric disorders are licensed for specific indications and vary across different countries. Yet, nearly one-third of adolescents (12–18 years old) diagnosed with depression consulted a child and adolescent psychiatrist, thus highlighting the gap in appropriate diagnosis, evidence, and therapeutic options for these young patients. The relative increase in antidepressant use was remarkable for adolescents (15–19-year-olds) from Denmark, Germany, and the United Kingdom; conversely, the same study found that the increase in antidepressant use was greatest among younger children (10–14-year-olds) in the Netherlands and the United States of America [8]. Despite government warnings, antidepressant use among children and adolescents has increased significantly in these five Western countries over the years [9], concluding that geographical variations exist regarding the usage of antidepressants for specific age groups of the population.

Adolescents are commonly treated with psychotherapy (60%), a combination of psychotherapy and antidepressants (10%), or only antidepressants (2%) [8]. Selective serotonin reuptake inhibitors (SSRI) were the most frequently prescribed antidepressants in Denmark (82% of all antidepressants), while tricyclic antidepressants (TCAs) were most commonly prescribed in Germany (23%) and the United Kingdom (19.5%) [9]. On the other hand, a survey conducted among children and adolescents from 2005–2012 (0–19 years) reported that the annual prevalence of antidepressant use rose from 1.3% to 1.6% in the United States of America, 0.7% to 1.1% in the United Kingdom, 0.6% to 1% in Denmark, 0.5% to 0.6% in the Netherlands, and 0.3% to 0.5% in Germany during the study period [8]. Adolescent girls (15–19 years) had higher utilization rates (12 to 14 per 1000 prescriptions) and accounted for most antidepressant use. Females were twice as likely as males to buy an antidepressant [10]. These rising figures emphasize the dire need to understand the effects of antidepressants, encompassing their associates/correlates, and their use among adolescents necessitates further research in this sphere.

The disparity across different age groups further expands into the severity of depression, co-morbidities, and other correlates that might affect the prescription patterns/trends. A study from Ontario also highlighted the systemic barriers to adhering to the integrated care pathway in adolescent depression, i.e., limited access to mental health expertise, long wait times for treatment, and a shortage of trained clinicians and evidence-based behavioral therapies [11]. Apparently, there is little information regarding the frequency and correlates of antidepressant prescriptions among adolescents in Germany. Therefore, this study aims to evaluate the cumulative incidence of antidepressant prescriptions, the types of antidepressant therapies administered among adolescents diagnosed with depression, and their associations with age, sex, and comorbidities in Germany. The results of this study might be able to guide clinicians and policymakers to revise the emergency pediatric and psychiatric practice guidelines or treatment modules as there are none [12].

## 2. Material and Method

### 2.1. Database

This retrospective cohort study used data from the Disease Analyzer^TM^ database (IQVIA), which has already been described in the scientific literature [13]. The database contains basic demographic information, diagnoses, and prescriptions collected from 2500 general and specialized practices in Germany. The Disease Analyzer^TM^ database selects practices based on multiple variables (i.e., German federal state, community size category, specialty group, and physician age). Finally, several studies on depression using data from this database have recently been published [13,14,15].

### 2.2. Study Population and Variables

**Figure 1:**
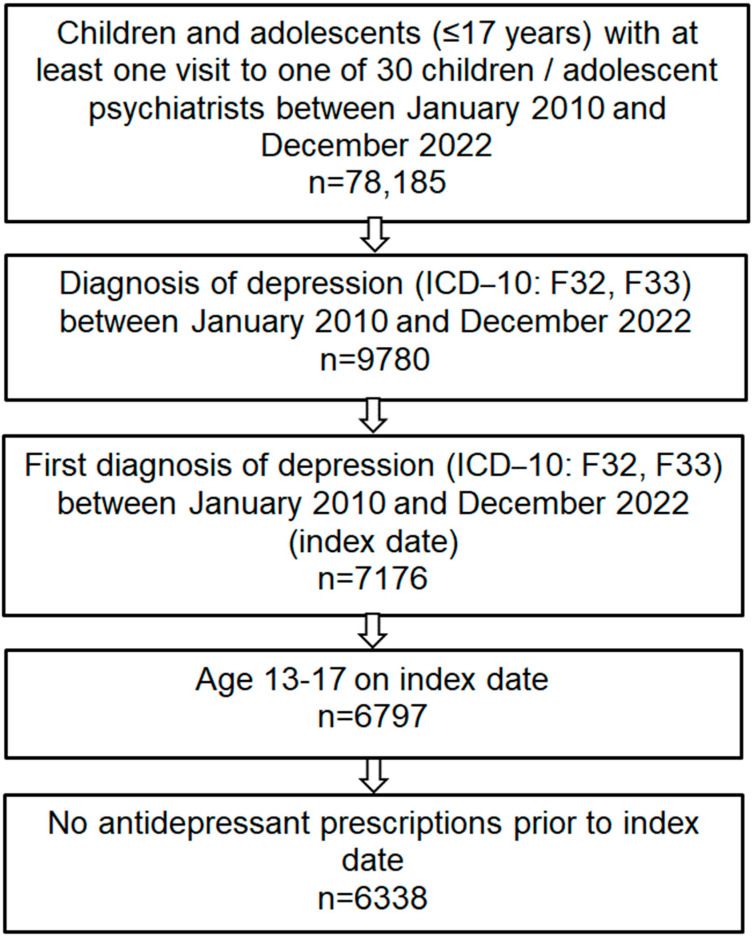
Flow chart of patients included in the study. Abbreviation: ICD–10 International Classification of Diseases, 10th revision.

This retrospective cohort study included adolescents aged 13–17 years diagnosed for the first time with depression (ICD-10 code: F32, F33) by one of 30 child and adolescent psychiatrists in Germany between 2010 and 2022 (index date). This study is limited to adolescents as depression in children under 13 years is a relatively rare disease, and the data source we used includes only small number of children (<13 years) with a depression diagnosis. The study flow chart is displayed in Figure 1. Depression severity included: mild depression (ICD-10 code: F32.0 F33.0), moderate depression (ICD-10 code: F32.1, F33.1), severe depression (ICD-10 code: F32.2, F32.3, F33.2, F33.3), and unspecified depression (ICD-10 code: F32.8, F32.9, F33.8, F33.9). The demographic variables studied were age and sex. Several chronic conditions frequently documented within 12 months before or on the index date included social phobias (ICD-10 code: F40.1), obsessive–compulsive disorder (OCD) (ICD-10 code: F42), post-traumatic stress disorder (ICD-10 code: 43.1), adjustment disorders (ICD-10 code: F43.2), eating disorders (ICD-10 code: F50), specific developmental disorders of scholastic skills (ICD-10 code: F81), attention-deficit hyperactivity disorders (ICD-10 code: F90), and emotional disorders with onset specific to childhood (ICD-10 code: F93).

### 2.3. Statistical Analyses

The baseline characteristics were described using absolute numbers (percentage) for all variables except for age, which was continuous and described using the mean value (standard deviation). The one-year cumulative incidence of antidepressant prescription (ATC: N06A) was studied using Kaplan–Meier curves and stratified by age, sex, and depression severity. In addition, the antidepressant classes (SSRIs, Serotonin, and Norepinephrine reuptake inhibitors (SNRI), TCAs, and herbal antidepressants) and the most frequently prescribed drugs were shown descriptively. Multivariable Cox regression analyses were conducted to assess the association between age, sex, depression severity, co-diagnosed conditions, and antidepressant drug prescription. The *p*-value of <0.05 was considered statistically significant. Analyses were performed using SAS version 9.4 (SAS Institute, Cary, NC, USA).

## 3. Result

### 3.1. Baseline Characteristics

The baseline characteristics of the 6338 patients included in this study are displayed in Table 1. The mean age was 15.7 ± 1.4 years; most patients were female (67.3%). The depression severities among adolescents were moderate (58.7%), undefined (15.9%), severe (12.9%), and mild (12.5%), in decreasing order of proportion. Finally, the three most commonly co-diagnosed emotional problems were adjustment disorders (20.8%), attention-deficit hyperactivity disorders (10.9%), and emotional disorders with onset specific to childhood (10.1%).

### 3.2. Cumulative Incidence of Antidepressant Prescription

**Figure 2:**
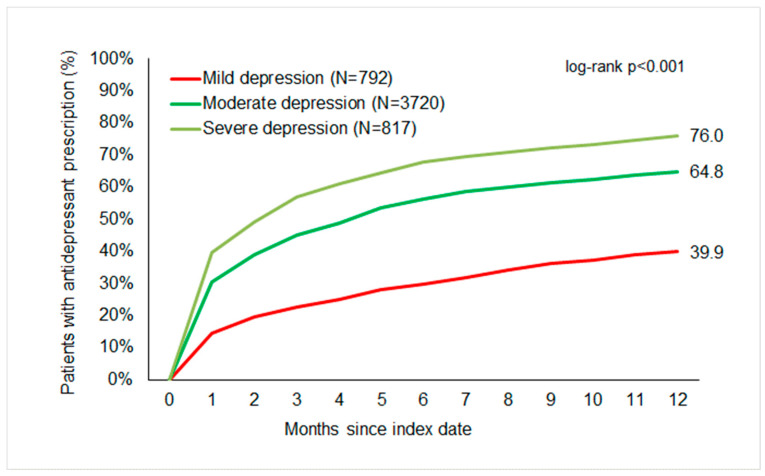
Cumulative incidence of antidepressant prescription in adolescents with depression by severity.

**Figure 3:**
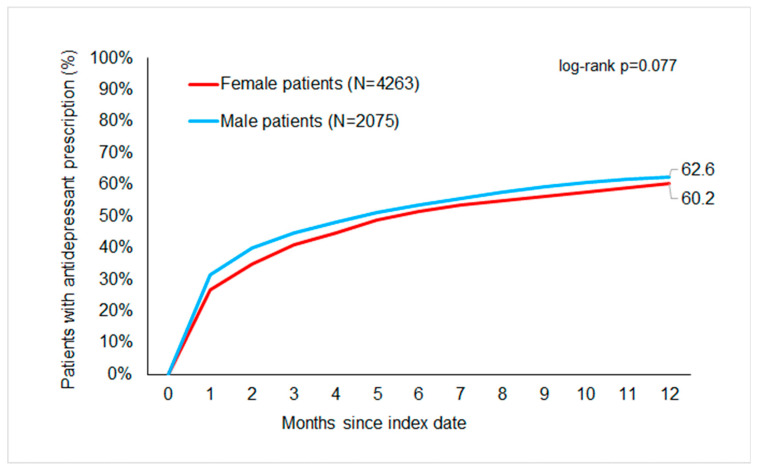
Cumulative incidence of antidepressant prescription in adolescents with depression by sex.

**Figure 4:**
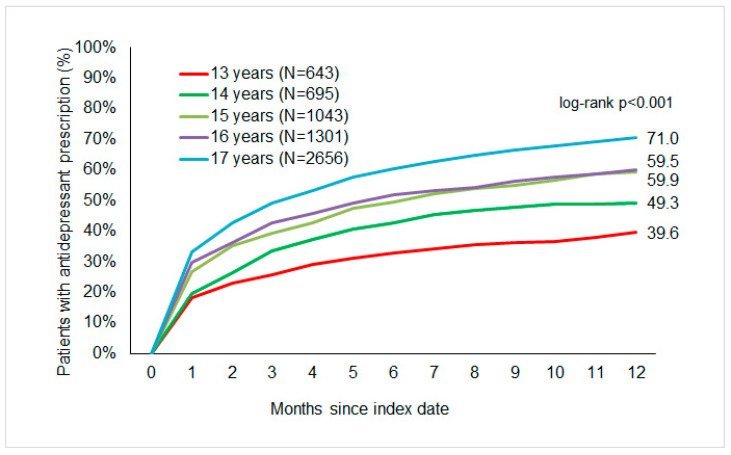
Cumulative incidence of antidepressant prescription in adolescents with depression by age.

**Figure 5:**
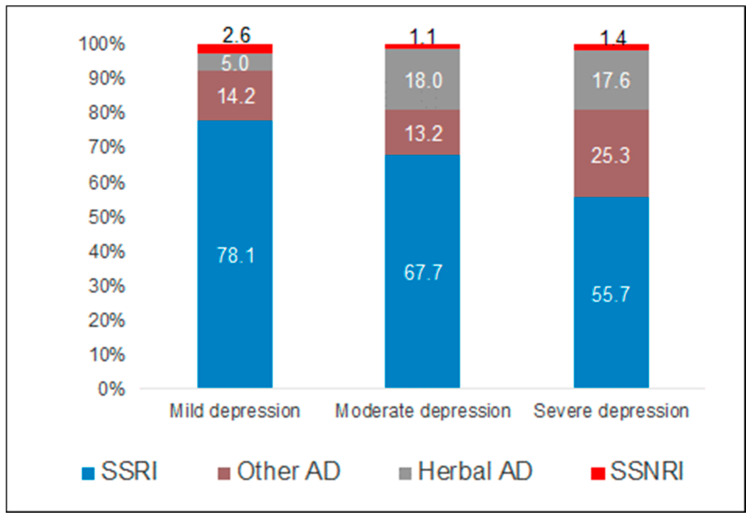
Distribution of antidepressant drug classes among adolescents with antidepressant prescriptions.

Figure 2 displays the cumulative incidence of antidepressant prescription by severity. The cumulative incidence of antidepressant prescriptions was 70%. About 39.9% of adolescents with mild, 64.8% with moderate, and 76% with severe depression received an antidepressant prescription within one year of the index date. However, most were treated within three months following the index date (Figure 3). No significant differences were observed in the proportion of antidepressant therapy between females (60.2%) and males (62.6%) (Figure 4). The cumulative incidence of antidepressant prescription increased with age from 39.6% in 13-year-olds to 71% in 17-year-olds (Figure 5).

### 3.3. Patterns of Prescription of Antidepressant Medications

Figure 5 shows the proportion of different antidepressant classes prescribed as first-line therapy within the first 12 months. SSRIs were the most frequently prescribed drug class for patients with mild (78.1%), moderate (67.7%), and severe (55.7%) depression. Where SSRIs were prescribed, Fluoxetine was the most common choice, followed by Sertraline and Escitalopram. SNRIs were rarely prescribed (~1%). Herbal medications (Hypericum Perforatum) were prescribed in 5% of patients with mild, 18% with moderate, and 17.6% with severe depression. Finally, the proportion of other drugs was 14.2% in patients with mild and 25.3% in those with severe depression. Of these drugs, only Mirtazapine, an atypical TCA, was prescribed in 65% of cases, while other medications were prescribed in insignificantly small proportions.

### 3.4. Factors Associated with the Prescription of Antidepressant Medications in the Year Following a Depression Diagnosis

The results of the multivariable logistic regression analyses are displayed in Table 2. Adolescents aged 13 years were compared with older adolescents, and it was observed that the risk of antidepressant prescription increased significantly at 14 years (HR: 1.23; 95% CI: 1.03–1.46), 15 years (HR: 1.5; 95% CI: 1.28–1.76), 16 years (HR: 1.53; 95% CI: 1.4–1.9), and 17 years (HR: 1.96; 95% CI: 1.7–2.26). Furthermore, a patient diagnosed with moderate (HR: 1.87; 95% CI: 1.65–2.11) or severe (HR: 2,29; 95% CI: 1.98–2.65) depression had a higher chance of being prescribed an antidepressant compared to patients with mild depression. Of the co-diagnoses examined, OCD (HR: 1.48; 95% CI: 1.29–1.7) and eating disorders (HR: 1.22; 95% CI: 1.06–1.4) were found to be associated with antidepressant prescriptions among the study population.

## 4. Discussion

The present study observed that the prevalence of antidepressant prescriptions within one year of the index date was greatest for adolescents suffering from severe depression, followed by those with moderate depression and mild depression. The cumulative incidence of antidepressant prescriptions increased with age and depression severity. SSRIs were the most frequently prescribed drug class among the study population, with Fluoxetine administered most commonly. Herbal medications were prescribed more frequently to those suffering from moderate depression or severe depression, with less frequent prescriptions for patients diagnosed with a milder form. The risk of antidepressant prescription increased significantly among older adolescents (>13 years old) and adolescents suffering from severe depression compared to those with mild depression. Lastly, OCD and eating disorders were found to be significantly associated with antidepressant prescriptions within the spectrum of co-diagnosed conditions.

Now, the relationship of depression with age is linear as per our findings; however, Bretschneider et al. highlighted a shift (between 2009 and 2012) in the age distribution of depression diagnoses in women and the severity of depression diagnosed among younger men, identifying an increase in the depression risk within specific sub-groups [3]. Further, there is also evidence of a consistent rise in antidepressant uses among adolescents (12–17 years) since 2005, mainly for SSRIs, although a decline was observed in children (5–11 years) for the same period [16]. Nevertheless, the variation in prescription by region and ethnicity could be due to certain inequities. The current evidence shows that antidepressant use among adolescents (13–17-year-olds) from Germany [8] has increased over time.

Ideally, moderate to severe depression should be treated with psychotherapy with or without Fluoxetine while treatment-resistant adolescents should receive a combination treatment with psychotherapy. Nevertheless, nil response to pharmacotherapy and psychotherapy may occur in nearly 40% of depressed adolescents. Studies should, therefore, explore the efficacy of antidepressants based on prevalent psychopathological dimensions (apathy, withdrawal, impulsivity, etc.) [17]. The results of this study are supported by previous researchers who reported that the antidepressants commonly prescribed were SSRIs (56%), Fluoxetine (24%), TCAs (18%), Citalopram (14%), Mirtazapine (10%), and St. John’s wort (9%) [8]. By contrast, an older study (2006) from Germany observed that the use of TCAs (80%) and SSRIs (15%) had doubled [10], which was not confirmed in our study. These differences might be due to changes in practice guidelines and the overall management of patients with depression. A meta-analysis (N = 17 RCTs) including 2537 children aged <18 years showed that antidepressants have significant positive effects on patients’ functioning but not on their quality of life. Furthermore, second-generation antidepressants (such as Fluoxetine, Escitalopram, and Nefazodone), unlike first-generation antidepressants, caused significant improvements in functioning in young patients with major depressive disorders [18]. Also, researchers suggest that newer antidepressants might help in reducing depressive symptoms in a small when compared with placebos or are insignificant in reducing depression symptoms. Especially, psychotherapy, in particular cognitive behavioral therapy, as per the country-specific or age-specific guidelines, remains important [7].

Herbal medicines generally comprise impurified plant extracts and tissues that are beneficial due to being better tolerated, having a lower cost, and having lesser side effects [19]. Further, some of these herbal medicines are available over the counter and are especially useful for treating patients with failed treatments or severe side effects from traditional drugs [20]. Despite their increasing popularity, herbal medicines in contrast to synthetic antidepressants, lack accurate concentrations, active ingredients, the exact biological chemicals, and are not tested for purity and potency with the same scientific rigor that is required for conventional drugs. Simultaneously, medicinal plants have a variability in pharmacologically active compounds, which might act on various potential targets, making it difficult to perform appropriate pharmacokinetic studies [21]. St. John’s wort products are approved as antidepressants by the German drug agency but not in the United States of America. Similar to our findings, previous researchers have found that adolescents were being prescribed herbal supplements as preferred self-medication because of their promising outcomes and the lower risk of side effects [22].

The management of major depression is concerning, with Fluoxetine currently being the only recommended pharmacological option. This is because it significantly improves symptom intensity and is well tolerated [23], as shown in a meta-analysis of seven trials. Likewise, our results reported that Fluoxetine was the most commonly prescribedSSRI. The tendency to prescribe Fluoxetine may be due to the lack of options for this age group, which is dependent on the intrinsic mechanisms mediating adolescent depression, i.e., the brain is in the development phase, causing pharmacological treatments to fail, achieve lower efficacy [24], or induce harmful responses [25,26]. By contrast, a recent review [7] showed that largely newer antidepressants may mildly reduce depressive symptoms when compared with placebo medication. An updated and expanded review [27] based on the previous version [7] mentioned that although Fluoxetine was the exclusive treatment option in previous reviews and the older (first) version of the German guidelines [28], there is now evidence that Sertraline, Escitalopram, and Duloxetine could have similar efficacy to Fluoxetine. Three RCTs among children (0–18 years) found Escitalopram to be effective, whereas two RCTs supported Citalopram for treating pediatric depression [29]. Similarly, an 8-week double-blind trial (N = 259) conducted in adolescents (aged 12–17 years) detected a significant improvement among patients treated with Escitalopram relative to those treated with the placebo. However, 10% of patients on Escitalopram reported headaches, menstrual cramps, insomnia, nausea, influenza-like symptoms, and even suicidality [26]. In addition, twenty-six RCTs on 6–18-year-olds indicated great uncertainty about the effects of Mirtazapine, Duloxetine, Vilazodone, Desvenlafaxine, Citalopram, or Vortioxetine on suicide-related outcomes compared to placebo. There is also a small amount of evidence that Escitalopram might “slightly” reduce the odds of suicide-related outcomes compared to placebo. However, Fluoxetine, Paroxetine, Sertraline, and Venlafaxine may “slightly” increase the odds of suicide-related outcomes compared to placebo. There is greater certainty that Venlafaxine “slightly” increases the odds of suicide-related outcomes compared to Desvenlafaxine and Escitalopram [7]. A review of 14 articles concluded that Sertraline is effective for depression among adolescents when combined with cognitive behavioral therapy [30]. The United States Food and Drug Administration has approved Escitalopram for treating adolescent depression, but there is insufficient evidence to support its approval yet [29].

A robust study determined the growth in the incidence of antidepressant prescriptions as 1.7 per 1000 minors in 2004 and 2.1 per 1000 minors in 2011. Depression was the leading diagnosis among all antidepressant users, and nearly one in four (36%) of all prescriptions were off-label in 2011 [31]. While drugs such as SSRIs and other antidepressants are not licensed for off-label use in children up to 17 years of age in Germany for the treatment of depressive disorders, Fluoxetine was approved in 2006. This large population-based cohort study (N = 13,035) also noted that nearly half (49%) of the participants were prescribed antidepressants off-label in Germany [32]. Nonetheless, a decline (from 58% to 41%) in off-label prescriptions was noted among individuals (<18 years) from 2004 to 2011. Although the off-label use of antidepressants in minors has decreased, it is still common, indicating a lack of approved drugs for managing depression for this age group rather than inappropriate medical treatment. Plus, off-label drug use has not been associated with an increased risk of adverse events compared to on-label use [33]. A recent prospective observational study, among hospitalized patients from three hospitals in Germany mentioned that usually, off-label drug use is performed for treating acute cases and emergencies as there is hardly any time for a clinician to reach out to a psychiatrist regarding the drug choices; thus, decisions are made based on previously acquired knowledge. Hence, the establishment of treatment protocols and training modules in healthcare institutes as per the national guidelines is mandatory [34]. Family/caregivers should closely monitor antidepressants prescribed to a child/adolescent as some of these medications may be associated with increased odds of suicide-related outcomes. Moreover, the ill effects of antidepressant drugs can be due to the heterogeneous nature of depression, and the response may be stronger in some patients [7].

A systematic review of 199 articles from North America and Europe published in the last two decades identified major depressive disorder as a significant risk factor for the development and the worsening of comorbidities (dementia, Alzheimer’s disease, cardiovascular disease, metabolic syndrome, autoimmune diseases, and drug abuse) [35]. Specifically, children and young adults are vulnerable to the development of anxiety disorders, depression, and eating disorders. Firstly, there is strong evidence that anxiety or depression in patients with eating disorders is significantly associated with the worsening of symptoms and prognosis, thus increasing the burden of illness. A double cross-sectional study from Germany witnessed a stark rise (26%) in eating disorders (bulimia and anorexia) among children (5–17 years) during the second lockdown of the COVID-19 pandemic [6]. Moreover, adolescent (12–25-year-old) females experienced higher impairment in anxiety/depression with worse eating disorder symptoms, and older children displayed greater impairment in mood dysregulation, self-esteem, and perfectionism as well as worsened eating disorder symptoms [36]. By comparison, the results of the present study reported that female sex and a diagnosis of an eating disorder increase the probability of antidepressant prescription. Consequentially, the severity of eating disorder symptoms is reported based on dysfunctional mood regulation, low self-esteem, and high levels of perfectionism, which are related to the development and maintenance of eating disorders. Thus, the results of the former study indicated a cumulative detrimental effect of co-occurring anxiety, depression, and eating disorders [36].

Also, a meta-analysis suggested that the benefits of antidepressants are high, especially for severe depression, and antidepressants are also used to treat anxiety disorders, obsessive–compulsive disorder, and post-traumatic stress disorder [37]. Pharmacotherapy with SSRIs and TCAs is the mainstay for treating individuals with OCD [38]. So, this might be the reason why patients with depression and OCD were found to be significantly more likely to be prescribed an antidepressant in our study. Furthermore, depression is the most common comorbidity in patients with OCD, and the severity of OCD symptoms is predictive of future depression across several years; however, depression symptoms are not predictive of the severity of future OCD symptoms. Therefore, treating OCD should be prioritized over the treatment of depression. In addition, as the risk of suicide is increased in patients with depression, continuous monitoring of depressive symptoms and suicidal ideation is required when treating OCD. There is also a possibility that patients with severe depression and lower motivation in performing daily activities may be hampered in their ability to engage in OCD treatment [39]. Hence, the treatment of adolescents with co-morbid conditions requires a multi-pronged approach. Particularly, cognitive behavioral therapy, pharmacotherapy (SSRIs, TCAs, SNRIs, anti-dopaminergic, glutamatergic, combination, etc.), deep brain stimulation, repetitive transcranial magnetic stimulation, and evidence-based therapy are given for improving the life of OCD sufferers [40].

The current study has several important strengths, including the diagnosis of depression by many practitioners across Germany with significant sample size and the longitudinal design. However, it is also subject to several limitations. Firstly, the sample was taken from an outpatient-based setting, so the findings may not be generalizable to hospital- or community-based populations. Similarly, the study results cannot be extrapolated to other countries. The database does not contain lifestyle-related factors or socioeconomic factors as well as information on differences within the country about the quality of care or modality of services offered, and a history of psychiatric illness in the family. Thirdly, as in other retrospective studies, conclusions can only be drawn about associations, not causal relationships. Moreover, cohort studies lack control over confounding variables. Lastly, a national-level telephone survey from Germany (N = 1009) examined the literacy of the public regarding depression and its severity levels, indicating a lack of knowledge that might adversely affect treatment adherence, especially for milder forms of depression [41]. This may be the reason why a reduced proportion of patients with mild depression reported having received an antidepressant prescription in the current study.

There are numerous practical and research implications of the present study. Firstly, our findings suggest that there is further scope for including patients with mild depression as the progression of the disease can be controlled at that stage. Additionally, studying newer antidepressants or herbal supplements and their effects on adolescents is vital, as currently the guidelines recommend Fluoxetine alone (NICE 2019) [42]. It is imperative to discuss the treatment options with children and their family members to closely monitor them for suicide-related outcomes, especially among children and adolescents living with comorbidities as trials largely exclude these children and adolescents [7]. So, routinely collected data may be useful to examine these cases over time. Secondly, our findings observed that even young children with depressive symptoms might present with other problems (OCD and eating disorders), so future longitudinal trials should incorporate children and adolescents and compare different subgroups for further analysis. With this context, meta-analyses of individual patient data might be useful in understanding whether the effects of different treatment schedules/drugs differ in particular subgroups. Lastly, a well-designed summarization of data on adverse effects of the disease/therapies, etc., and further investigations among young children would be helpful.

In spite of depression being a major public health issue, and despite the availability of numerous pharmacological and non-pharmacological therapies, many patients suffering from depressive symptoms remain undiagnosed or receive inappropriate treatment. Policymakers could combine existing drug therapies with feasible and cost-effective non-pharmacological alternatives. Additionally, robust awareness programs can reduce the gaps in treatment, and dissemination of effective and lifesaving life skills might aid in maintaining positive mental health in the community [12]. Improved quality of integrated care involving primary care and mental health clinicians might help in identifying and treating adolescent depression early [11]. The literature highlights the need to address depression at an early age and integrate mental and general health care into pediatric practice. Further research should examine prescribing trends, risk factors of antidepression medications, access to diagnosis or treatment, prescribing behavior, country-specific prescription guidelines, or help-seeking behavior among young people. Moreover, other pharmacological and non-pharmacological interventions should be studied for depression in this age group as currently only one drug is approved [24]. More studies should emphasize the actual burden and the therapeutic options available for patients with mild depression to halt its progression. Policymakers should consider recommending the use of newer-generation antidepressants for some individuals under specific circumstances [7].

## 5. Conclusions

In this large retrospective study, the majority of adolescents with depression diagnosis received an antidepressant prescription. The chance of antidepressant prescription increases significantly with age, depression severity, and co-diagnoses like OCD or eating disorders. Fluoxetine was the most frequently prescribed drug for depression. Finally, based on these findings, there is a need for more data to corroborate or invalidate our results in other countries.

## Figures and Tables

**Table 1:** Baseline characteristics of the study sample.

Variable	Patients with Depression (N = 6338)
Age (in years)	
Mean (standard deviation)	15.7 (1.4)
13 years	643 (10.2)
14 years	695 (11.0)
15 years	1043 (16.5)
16 years	1301 (20.5)
17 years	2656 (41.9)
Sex	
Female	4263 (67.3)
Male	2075 (32.7)
Depression severity	
Mild	792 (12.5)
Moderate	3720 (58.7)
Severe	817 (12.9)
Other or undefined	1009 (15.9)
Co-diagnoses documented within 12 months prior to or on the index date	
Social phobias	452 (7.1)
Obsessive-compulsive disorder	371 (5.9)
Post-traumatic stress disorder	620 (9.8)
Adjustment disorders	1318 (20.8)
Eating disorders	429 (6.8)
Specific developmental disorders of scholastic skills	332 (5.2)
Attention-deficit hyperactivity disorders	690 (10.9)
Emotional disorders with onset specific to childhood	637 (10.1)

Data are absolute numbers (percentage) unless otherwise specified.

**Table 2:** Variables associated with the probability of antidepressant prescription in adolescents with depression diagnosis.

Variable	Hazard Ratio (95% CI)	*p*-Value
Age		
13 years	Reference	
14 years	1.23 (1.03–1.46)	0.023
15 years	1.50 (1.28–1.76)	<0.001
16 years	1.63 (1.40–1.90)	<0.001
17 years	1.96 (1.70–2.26)	<0.001
Sex		
Female	Reference	
Male	1.01 (0.93–1.09)	0.826
Depression severity		
Mild	Reference	
Moderate	1.87 (1.65–2.11)	<0.001
Severe	2.29 (1.98–2.65)	<0.001
Co-diagnoses documented within 12 months before or at the index date		
Social phobias	1.14 (1.00–1.29)	0.057
Obsessive–compulsive disorder	1.48 (1.29–1.70)	<0.001
Post-traumatic stress disorder	1.13 (1.00–1.27)	0.051
Adjustment disorders	1.08 (0.99–1.18)	0.105
Eating disorders	1.22 (1.06–1.40)	0.006
Specific developmental disorders of scholastic skills	0.93 (0.78–1.09)	0.353
Attention-deficit hyperactivity disorders	0.95 (0.84–1.06)	0.343
Emotional disorders with onset specific to childhood	0.92 (0.82–1.05)	0.206

## Data Availability

The datasets used and analyzed during the current study are available from the corresponding author upon reasonable request due to privacy restrictions.
